# Target and peripheral dose during patient repositioning with the Gamma Knife automatic positioning system (APS) device

**DOI:** 10.1120/jacmp.v11i1.3150

**Published:** 2010-01-28

**Authors:** Tuan‐Anh Tran, Thomas Stanley, Harish K. Malhotra, Steven F. deBoer, Dheerendra Prasad, Matthew B. Podgorsak

**Affiliations:** ^1^ Department of Radiation Medicine Roswell Park Cancer Institute Buffalo NY USA; ^2^ Department of Molecular and Cellular Biophysics and Biochemistry Roswell Park Cancer Institute Buffalo NY USA; ^3^ Department of Physiology and Biophysics State University of New York at Buffalo Buffalo NY USA

**Keywords:** Leksell Gamma Knife, automatic positioning system, stereotactic radiosurgery, treatment planning system, dose distribution

## Abstract

The GammaPlan treatment planning system does not account for the leakage and scatter dose during APS repositioning. In this study, the dose delivered to the target site and its periphery from the defocus stage and intershot couch transit (couch motion from the focus to defocus position and back) associated with APS repositioning are measured for the Gamma Knife model 4C. A stereotactic head‐frame was attached to a Leksell 16 cm diameter spherical phantom with a calibrated ion chamber at its center. Using a fiducial box, CT images of the phantom were acquired and registered in the GammaPlan treatment planning system to determine the coordinates of the target (center of the phantom). An absorbed dose of 10 Gy to the 50% isodose line was prescribed to the target site for all measurements. Plans were generated for the 8, 14 and 18 mm collimator helmets to determine the relationship of measured dose to the number of repositions of the APS system and to the helmet size. The target coordinate was identical throughout entire study and there was no movement of the APS between various shots. This allowed for measurement of intershot transit dose at the target site and its periphery. The couch was paused in the defocus position, allowing defocus dose measurements at the intracranial target and periphery. Measured dose increases with frequency of repositioning and with helmet collimator size. During couch transit, the target receives more dose than peripheral regions; however, in the defocus position, the greatest dose is superior to the target site. The automatic positioning system for the Leksell Gamma Knife model 4C results in an additional dose of up to 3.87±0.07%,4.97±0.04%, and 5.71±0.07% to the target site; its periphery receives additional dose that varies depending on its position relative to the target. There is also dose contribution to the patient in the defocus position, where the APS repositions the patient from one treatment coordinate to another. This may be important for treatment areas around critical structures within the brain. Further characterization of the defocus and transit exposures and development of a dose calculation algorithm to account for these doses would improve the accuracy of the delivered plan.

PACS numbers: 87.53.‐j, 87.53.Bn, 87.53.Dq, 87.53.Ly

## I. INTRODUCTION

The Gamma Knife model 4C is a highly precise stereotactic radiosurgery tool for the treatment of intracranial lesions.^(^ ^1^ ^–^ ^4^ ^)^ Arranged in five parallel‐plane rings forming a portion of a hemisphere, 201 individual C60o sources emit gamma radiation focused at a single isocenter. The size of the beamlets from each source can be approximately 4, 8, 14 or 18 mm in diameter at the isocenter, depending on the helmet used.^(1)^

The Gamma Knife model 4C has an automatic positioning system (APS) used to reposition the patient's head to different coordinates in a treatment run.^(^ ^2^ ^–^ ^3^ ^,^ ^5^ ^)^ During patient repositioning, the couch moves out of the docking (or treatment) position to the defocus position (28 cm away) where the APS moves to the coordinates of the next shot.^(5)^ Once the patient's head is properly positioned, the couch moves back into the machine and into treatment position to allow delivery of dose to the target site. The process is repeated until all shots within a run are delivered. Subsequent runs, if any, are delivered in a similar process.

There are three sources of undocumented doses during a typical treatment: the transportation dose, the intershot transit dose (or transit dose), and the defocus dose. The transportation dose results from the exposure the patient receives while moving between the setup and treatment positions at the beginning and end of a run; the shielding doors are open and the patient is exposed to the scatter radiation from the sources.^(^ ^6^ ^–^ ^8^ ^)^ The transit dose results from exposure when the couch moves between the focus and defocus positions. Again, the shielding doors are open as the couch moves away from the focal point of the 201 beamlets and into a defocus position, where the APS can transition between shot coordinates. Finally, while in the defocus position, exposure results from leakage and scatter from the unshielded sources.

There is considerable emphasis on conformal dose planning to improve conformity indices as a dosimetric quality measure of a radiosurgery treatment plan. This often compels the use of multiple isocenters which require multiple repositioning by the APS; the consequence, however, is the potential for significant, undocumented dose. This can be a concern when treating near critical structures as this additional dose to these anatomical structures may not be fully accounted for during planning. Currently, the treatment planning system does not incorporate transportation, intershot transit, and defocus doses. In this work, we report the intracranial dose while the patient is in the defocus position and during couch intershot transit. We look at the dose to the target site and its periphery, and map the distribution of additional intracranial dose from APS repositioning in the defocus and transit positions.

## II. MATERIALS AND METHODS

### A. Setup

**Figure 1:**
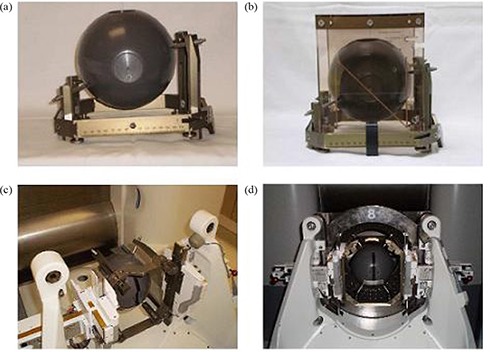
Setup of phantom for measurements: (a) framed phantom, (b) framed phantom with CT fiducial box for imaging, (c) and (d) framed spherical phantom placed in APS.

During this study, the dose rate at the center of a spherical phantom (Elekta Corporation, Atlanta, GA) with the 18 mm helmet ranged from 2.256 to 2.190 Gy per minute. The phantom was framed with a standard Elekta stereotactic frame (Leksell Coordinate Frame G, Elekta, Atlanta, GA) and imaged with a GE HiSpeed FX/i CT scanner (GE Healthcare, Waukesha, WI) (Fig. 1(a)). A CT fiducial box was placed onto the frame while a scan with 1 mm slice thickness was acquired (Fig. 1(b)); images were exported to and registered in the GammaPlan (Version 8.2, Elekta, Atlanta, GA) treatment planning system. The center of the target was selected to be the center of the spherical phantom with a dose prescription of 10 Gy to the 50% isodose line. The framed phantom was coupled to the APS (Fig. 1(c) and 1(d)) for dose measurements. Within the phantom, a calibrated 0.06 cm^3^ cylindrical ionization chamber (model PR‐05P, Capintec, Ramsey, NJ) was positioned using a chamber cassette and connected to an electrometer (35617EBS Programmable Dosimeter, Keithley Instruments, Cleveland, Ohio). All measurements were repeated multiple times to enable statistical analysis.

### B. Transit dose to target

Measurements were taken for single run plans with varying numbers of shots (1, 5, 10, 20, 35, and 50) with the 8, 14 and 18 mm helmets (the 4 mm helmet was not measured due to concerns with reliability of measurements). Multiple shot plans were generated to allow measurements of transit dose accumulated during the transition between the focus and defocus positions. The plans were designed such that no physical repositioning with the APS occurred – the treatment coordinates remained the same throughout the measurements. The measured doses may be the minimum that a patient would receive during the process of APS repositioning for a given number of shots because there was no change in coordinates. Each run was timed with a stopwatch; the time difference between the single shot run and the multiple shot run is the transit time. The differences between the dose measured for the single shot run and the multiple shot runs for the same prescription dose represented the transit dose to the target site from the couch transit. The average transit dose rate is calculated by taking the total transit dose and dividing by the corresponding transit time to give the average transit dose rate. This experimental design eliminates transportation dose because all plans result in equal transport from the setup position to the focus position and back.

### C. Transit dose to periphery

Peripheral dose was measured to determine dose to normal tissue, or nontarget sites within the cranium. The spherical phantom had to be framed twice with specific orientations to measure dose along the x‐axis (lateral) and the z‐axis (cranial‐caudal). To measure the dose along the x‐axis, the cassette with the insert for the ionization chamber was aligned in the transverse plane and the direction of the cylindrical cavity within the cassette was aligned along the x‐axis. Measurements were taken at 0,±1.2,±3.2 and ±6.2cm from isocenter along the x‐axis for the 1, 5, 20 and 50 shot run plans to determine the transit dose. Measurements along the z‐axis require the cylindrical cavity to be aligned along the couch longitudinal axis. Along the z‐axis, 1, 5 and 20 shot runs were measured at 0,+1.2,±3.2 and ±6.2cm to determine the transit dose at each peripheral position. The same method of calculating average transit dose rates at the target is used to determine the values at off‐axis locations.

The ionization chamber was repositioned along the cylindrical cavity of the fixed spherical phantom, from the center to 6.2 cm from the center. Cylindrical Lucite rods (1, 2 and 3 cm long) of 6 mm diameter were created to position the chamber off‐axis. The rods were inserted followed by the chamber, displacing the chamber from the phantom center by the length of the rod(s). With these rods, the chamber can be positioned to 1.2, 3.2 and 6.2 cm from the center of the phantom. The additional 0.2 cm comes from the incomplete insertion of the cylindrical rods at the end of the cavity.

### D. Dose in the defocus position

To measure the defocus dose (in the defocus position where the APS changes shot coordinates), a plan was generated with multiple shots to the center of the spherical phantom. As with peripheral measurements during couch transit, the phantom was framed twice to measure dose along the x‐ and z‐axes. Clinically, the time for the APS to move from one treatment position to another varies and will depend on the distance between the shot coordinates. In order to have enough time to measure the defocus dose, the “Emergency Stop” button was pressed to suspend all system motions (APS, couch, shielding door, etc) once the couch reached defocus position. The shielding doors remained open as they would during patient repositioning by the APS, and the couch remained in the defocus state simulating repositioning from one shot to the next. In the defocus position, measurements were taken at 0,±1.2,±3.2 and ±6.2cm from isocenter along both x‐ and z‐axes. To measure defocus dose, the electrometer was programmed to record the cumulative charge collected at one minute time interval. Readings collected after the couch reached the defocus position were analyzed. The defocus dose rates were determined directly from the collected charge readings (readings were taken every minute and charge was converted to dose to give dose rates).

## III. RESULTS

### A. Transit dose to target

**Figure 2:**
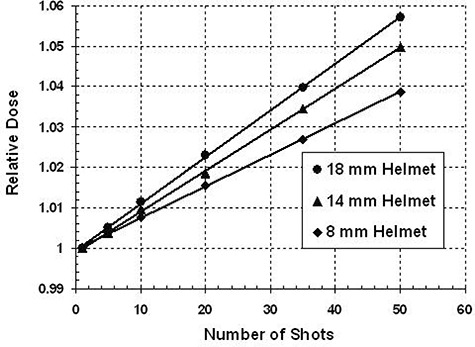
Relative dose as a function of number of shots for the 8, 14 and 18 mm helmets. The target position is the same for all measurements. The relative dose was calculated by taking the ratio of all multiple shot plans to the single shot plan. Measured dose from APS repositioning increases with increase in the number of shots in a run; it also increases with helmet size.

The transit dose increases with frequency of repositioning and helmet collimator size (Fig. 2). Using 50 shots within one run, the total delivered dose can be up to 3.87±0.07%,4.97±0.04%, and 5.71±0.07% higher than the planned dose as a result of leakage and scatter during couch transit for APS repositioning with the 8, 14, and 18 mm helmets, respectively. Therefore, dose increases of 0.08% (or 1.48±0.03cGy), 0.10% (or 1.97±0.02cGy), and 0.12% (or 2.27±0.03cGy) are expected per reposition for the 8, 14 and 18 mm helmets, respectively. In the extreme case of a 50‐shot plan, this represents 72.5 cGy, 96.5 cGy and 111.0 cGy of extra dose to the patient, as compared to the entire dose being delivered in a single shot. The average transit dose rate for each of the helmets used is listed in Table 1 along with its value relative to the focus dose rate for the day of measurement (2.25 Gy per minute for the 18 mm helmet).

**Table 1:** Average transit dose rates at the target position. The average transit dose rate increases with increase in helmet size. The average transit dose rate is represented as a percent of the focus dose rate on the day of measurements (2.25 Gy per minute for 18 mm helmet).

	*Helmet Size and Average Transit Dose Rates*	
*Helmet Size (mm)*	*Measured Transit Dose Rate (cGy/min)*	*Percent of Focus Dose Rate (%)*
18	8.81±0.41	3.91±0.18
14	6.98±0.51	3.10±0.23
8	5.89±0.51	2.62±0.23

### B. Transit dose to periphery

**Figure 3:**
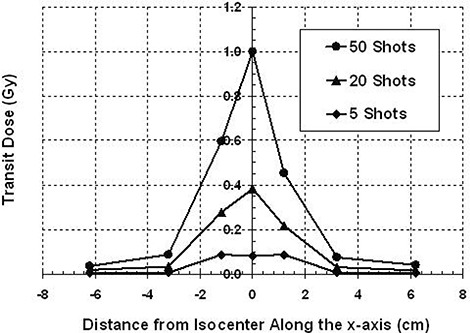
Transit dose along the x‐axis for the 18 mm helmet. The transit dose is greatest at isocenter and falls off with distance. Transit dose will increase with the number of shots.

Figure 3 shows a plot of the transit dose along the x‐axis. There are three separate plots; each represents a different number of shots in a single run. The greatest transit dose is at the target site and falls off symmetrically with increasing distance from the isocenter. Transit dose to the periphery increases with increasing number of shots in a run, as is the case with transit dose to the target site. The average transit dose per reposition at the target is 2.05±0.03cGy and it falls off symmetrically with distance along the x‐axis. Table 2 gives the average transit dose rates along the x‐axis. Also listed are the percent dose rates (average transit dose rate as a function of the dose rate at the focus point on the day of measurement, which is 2.22 Gy per minute for the 18 mm helmet). These dose rate values may be applicable to other centers.

**Table 2:** Average transit dose rates along the x‐ and z‐axes. The average transit dose rate is also represented as a percentage of the focus dose on the day of measurement rate (2.22 Gy per minute for the 18 mm helmet). The difference between the doses measured at 0 cm along the x‐ and z‐axes results from the nonisotropic isodose distribution at isocenter; dose measured will depend on the orientation of the ionization chamber.

*Average Transit Dose Rates Along the x‐axis*		
*Position (cm)*	*Measured Transit Dose Rate (cGy/min)*	*Percent of Focus Dose Rate (%)*
−6.2	0.36±0.06	0.16±0.03
−3.2	0.70±0.04	0.32±0.02
−1.2	6.11±1.85	2.75±0.83
0	7.72±0.08	3.48±0.04
1.2	5.28±2.45	2.38±1.10
3.2	0.59±0.02	0.27±0.01
6.2	0.36±0.04	0.16±0.02
*Average Transit Dose Rates Along the z‐axis*		
*Position (cm)*	*Measured Transit Dose Rate (cGy/min)*	*Percent of Focus Dose Rate (%)*
−6.2	0.00±0.00	0.00±0.00
−3.2	0.10±0.10	0.05±0.05
0	8.66±0.19	3.90±0.08
1.2	1.37±0.30	0.62±0.14
3.2	1.88±0.14	0.85±0.06
6.2	3.34±0.04	1.51±0.02

**Figure 4:**
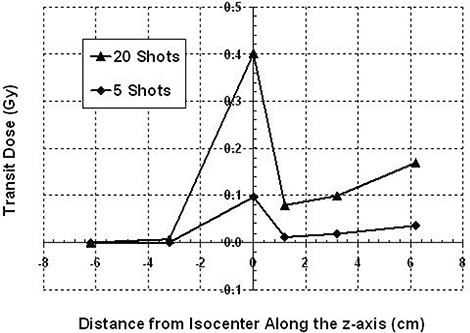
Transit dose along the z‐axis for the 18 mm helmet. The transit dose is greatest at isocenter; however, dose will increase in more superior regions of the sphere phantom. This increase can be attributed to the lack of sufficiently attenuating material at the crown of the helmet as the helmet cap is 8 mm of fiberglass with a 5 cm hole at its apex.

**Figure 5:**
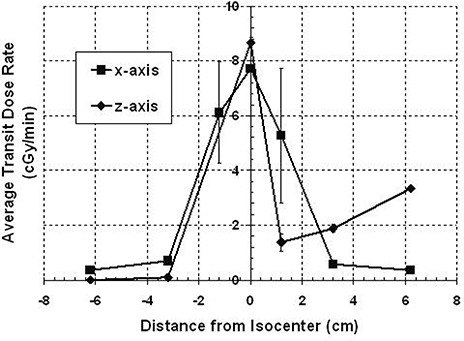
A comparison of the average transit dose rates along the x‐ and z‐axes for the 18 mm helmet. Dose rates are represented as a percent of the focus dose rate. The difference in dose rates at isocenter may be due to the orientation of the ion chamber for each measurement.

Along the z‐axis there is not the same symmetry in transit dose as seen with dose measurements along the x‐axis (Fig. 4). The dose is still larger at the target site and falls off inferiorly; however, superior to the target site, after initially decreasing, the transit dose increases. The transit dose per reposition is 2.26 cGy for the target; at 6.2 cm superior to the target, it is 0.90 cGy per reposition and at 6.2 cm inferior to the target, the measured transit dose is 0.003 cGy per reposition. Table 2 lists the average transit dose rates along the z‐axis. Also presented is the percent of the focus dose rate on the day of measurement (2.22 Gy per minute for the 18 mm helmet). The average transit dose rate at superior regions of the phantom can be up to 3.34±0.04cGy per minute. The average transit dose rates were calculated and plotted for positions on both axes (Fig. 5). The discrepancy in transit doses at isocenter is the result of the nonisotropic isodose distribution at isocenter, where the dose is weighted more in the superior direction. When the ion chamber is placed at isocenter, the exposure integrated over the collecting volume will be greater with the ion chamber oriented in the z‐direction than if oriented along the x‐direction. The error bars at the ±1.2cm positions along the x‐axis are relatively large, possibly resulting from the high dose gradient at these locations for the 18 mm helmet.

### C. Defocus dose to target and periphery

**Figure 6:**
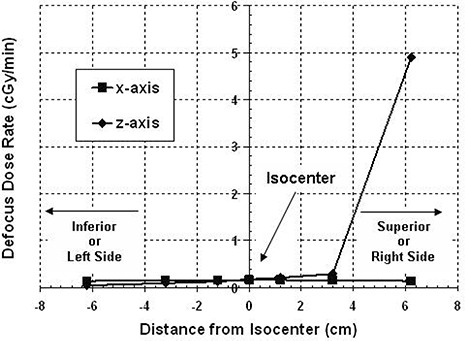
The dose rate at the couch defocus position for the 18 mm helmet. The defocus dose is low along the x‐axis (<0.160±0.005cGy/min) and most of the z‐axis. In the more superior regions, the defocus dose rate is greater (4.910±0.005cGy/min at 6.2 cm superior to the isocenter). Defocus dose rate is expected to be higher with proximity to the superior surface of the spherical phantom. Proximity of the phantom to the crest of the helmet will also have an expectation of greater exposure due to the inverse square law (this will occur for treatment areas that are inferiorly located).

Couch transit time between the focus and defocus positions is fixed, but time in the defocus position depends on the intershot distance. Therefore, unplanned dose may have a greater contribution from either the couch defocus or transit positions, depending on the time for the APS to reposition from one shot coordinate to the next. In the defocus position, the measured dose rate is significant at more superior regions (Fig. 6). The dose rates measured along the x‐axis are small and comparable (0.15±0.01cGy per minute). Along the z‐axis, the dose rate is also small, except at the most superior location measured (Fig. 4). At +6.2cm, the defocus dose rate is 4.91±0.01cGy per minute, which is more than the average transit dose rate measured at the same position (3.34±0.04cGy per minute).

## IV. DISCUSSION

### A. Measured data

Couch transit for repositioning of the patient's head by the APS results in additional dose to the target site and its periphery. The intershot transit dose increases with the number of shots required in a run and increases with collimator size. Because the plans were designed such that no physical repositioning with the APS system occurred, the measured doses may be the minimum that a patient would receive during the process of APS repositioning for a given number of shots. The average transit dose rate may be higher or lower, depending on the activity of the sources. It may also depend on the time it takes for the couch to go from the focus to the defocus position and back (16 seconds for our machine). In an actual patient treatment, the unaccounted dose will also depend upon the time needed for the APS to transit between treatment coordinates. The treatment planning system limits the number of shots in a run to 50; if more shots and runs are required to obtain conformity or adequate dose to the lesion, the unaccounted dose may actually be more than reported here.

The transit dose will contribute more to the target and its margin than the defocus dose, as shown by the data. Because the shape of the dose distribution follows the shape of the beam profile at isocenter, it is reasonable to conclude that the transit dose contribution to the target is the result of the partial alignment of the primary and secondary collimators just before docking and after undocking between the helmet and sources. This distribution is not seen with measurements in the couch defocus position, suggesting minimal dose coming through the secondary collimators here. Many shots in a run may risk unintended overexposure of surrounding normal tissue from transit dose because the GammaPlan software does not account for the intershot transit dose from APS repositioning. This is especially true for plans with larger helmet sizes and greater number of repositions.

Greater dose rates are expected at more superior regions within the phantom because of proximity to the sources. Also, as the helmet moves away from and towards the sources, the unfocused beam will intersect the fiberglass helmet cap (where there is little attenuation of the beam) exposing the superior region of the phantom or patient to unintended radiation. The transit dose increases at positions closer to the crown of the head because of the poorly shielded 23 cm diameter opening at the helmet's apex, which results in greater exposure from leakage and scatter at superior regions.^(9)^ This is the reason for the behavior of the increased transit dose along the superior portion of the z‐axis (Fig. 4). In the defocus position, measured dose is likely from the scatter and leakage through the 23 cm diameter opening and will be independent of the helmet collimator size.^(10)^

The amount of peripheral exposure during repositioning will depend on location. Superior regions receive significant dose from both the transit and defocus positions; however, inferior positions receive dose mainly from the transit. In the peripheral region of the target along the x‐axis, unfocused dose contribution comes mainly from the couch transit. However, the more superior the z‐plane is placed to measure peripheral dose along the x‐axis, the more the dose contribution will be dominated by the defocus dose. The greater superior dose contribution from the defocus position was unexpected. One expects that unfocused dose would be related to the proximity to the sources, and since all points along the couch transit path are closer to the sources for the phantom than when the couch is in the defocus position, the measured dose rate should be higher with couch transit. However, the data show that the rate of scatter radiation to superior areas of the phantom is greater in the defocus position than during couch transit (Figs. 5 and 6).

### B. Previous studies

Earlier Gamma Knife models (U and B2) did not have an APS device and head shielding designs were different. To change from one coordinate position to another, the couch would need to move from the focus to the setup position where coordinates would be changed manually; the system is inherently limited to one shot per run. Ertl et al.^(7)^ performed a study with the model B2 to look at the differences between a single run plan and multiple run plans of the same total dose prescription. The dose difference between the single run plan and 50 run plan for the 18 mm helmet was 10 Gy (focus dose rate was 3.005 Gy per minute).^(7)^ This is about nine times more than the dose we measured using the APS (1.11±0.01Gy with a focus dose rate of 2.190 to 2.256 Gy per minute). The modifications from the model B to the model C Gamma Knife greatly reduced unplanned dose to the patient.

With the model C Gamma Knife using an APS, Bradford et al.^(9)^ studied unaccounted dose at regions close to the scalp of the head to account for the cause of epilation. They found that the dose increases with proximity to the helmet cap – the transit dose increases with positions superior to the focus. Though the technique they used to measure defocus dose was different, our measured data is consistent with their values at more superior locations for defocus dose. The most superior measurement we took was at 6.2 cm from the focus, where the average transit dose rate was 3.34 cGy per minute and the defocus dose rate was 4.91 cGy per minute. The authors note that there is a sharp rise in dose at the 6 cm position; the dose they measured was 5.6 cGy per shot.^(9)^ We also found a sharp increase in defocus dose and a rise in transit dose at this position (positions beyond this were not studied). Bradford et al. attributed the high dose at the superior region of the head to the low attenuation of the unfocused beam by the 0.8 cm thick fiberglass helmet cap as the couch moves in and out of focus position for repositioning.^(9)^

Watanabe et al.^(10)^ studied dose at the defocus position, where the APS changes shot coordinates. They found that defocus dose is independent of both helmet size and position of the shot within the phantom relative to the measurement point.^(10)^ This is consistent with our observations because in the defocus position, most of the dose is entering through the helmet cap region at the apex of the helmet. The level of exposure within the phantom will vary in the defocus position; in a given x‐y plane, the dose will be similar, but will change with transverse position. There is dose contribution to the patient in the defocus position, but it is mostly negligible as discussed by Watanabe et al. However, defocus dose in superior regions can be considerable, especially when there are multiple shots in a single run and when the treatment volume is located inferiorly.^(^ ^9^ ^–^ ^10^ ^)^

## V. CONCLUSIONS

For multiple shot runs, use of the APS device results in additional unaccounted dose to the target site and its periphery. Previously unreported and uncharacterized are the relationship between unaccounted dose and helmet size, transit dose and number of repositions, and the positional dependence of dose to the focus. Though regarded as one of the most accurate modality for intracranial radiosurgery, there is still potential for substantial unaccounted dose during treatment resulting from APS repositioning. This may be important for treatment regions around critical structures within the brain. Further characterization of the defocus and transit exposures and development of a dose calculation algorithm to account for these doses would improve the accuracy of delivered dose.

## ACKNOWLEDGEMENTS

Tuan‐Anh Tran wishes to acknowledge financial scholarship support from the Gates Millennium Scholars Program. The authors would like to thank Mary Elizabeth Jurca RN, Jennifer Buil RTT, Joyce Martin RTT, and Gail Thompson RTT; their assistance with this project is greatly appreciated. We also thank Lalith Kumaraswamy MS for his help with the analysis, and all the members of the Roswell Park Cancer Institute Gamma Knife Team for their support.
